# Advancing Insights Into Visceral Leishmaniasis: Challenges, Innovations, and Future Directions in Global Disease Management

**DOI:** 10.1155/japr/5233179

**Published:** 2025-12-30

**Authors:** Ebrahim Abbasi

**Affiliations:** ^1^ Research Center for Health Sciences, Institute of Health, Shiraz University of Medical Sciences, Shiraz, Iran, sums.ac.ir; ^2^ Department of Medical Entomology and Vector Control, School of Health, Shiraz University of Medical Sciences, Shiraz, Iran, sums.ac.ir

**Keywords:** diagnostics, HIV co-infection, kala-azar, *Leishmania donovani*, neglected tropical diseases, therapeutics, vector control, visceral leishmaniasis

## Abstract

**Background:**

Visceral leishmaniasis (VL), also known as kala‐azar, is a life‐threatening neglected tropical disease primarily caused by *Leishmania donovani* and transmitted by infected sandflies. Despite notable advancements in public health, VL continues to pose significant challenges, especially in South Asia, East Africa, and South America.

**Methods:**

A systematic review was conducted in accordance with PRISMA guidelines. Peer‐reviewed literature published from 2000 to 2024 was retrieved from PubMed, Scopus, and Web of Science to assess epidemiological patterns, diagnostic advancements, therapeutic options, and vector control strategies.

**Results:**

The analysis revealed progress in diagnostic tools such as rK39‐based rapid tests and molecular diagnostics. Therapeutic improvements, notably liposomal amphotericin B and miltefosine, have emerged, although drug resistance and limited accessibility remain problematic. Vector control through residual spraying and community programs shows promise but is hindered by insecticide resistance. Furthermore, HIV co‐infection and climate‐driven spread exacerbate control efforts.

**Conclusions:**

Comprehensive management of VL requires integrative approaches that combine novel diagnostics, targeted treatment, innovative vector control, and robust public health systems. Emphasis on vaccine development, digital health solutions, and community participation is crucial for sustainable control and eventual elimination.

## 1. Introduction

Visceral leishmaniasis (VL), also known as kala‐azar, remains one of the most severe and neglected tropical diseases, affecting millions of individuals across endemic regions in South Asia, East Africa, and South America. Despite significant advancements in public health interventions and disease control strategies, the burden of VL continues to disproportionately impact vulnerable populations, particularly in resource‐limited settings where health infrastructure is inadequate. The disease is caused by protozoan parasites of the *Leishmania donovani* complex, transmitted through the bites of infected female *Phlebotomus* or *Lutzomyia* sandflies. Characterized by prolonged fever, hepatosplenomegaly, severe weight loss, anemia, and immunosuppression, VL poses a significant challenge to health systems due to its high mortality rate if left untreated. Vector control, including residual spraying, insecticide‐treated nets, and community‐based interventions, remains a key pillar of VL management [1–3].

The global distribution of VL reflects complex epidemiological patterns influenced by environmental, socioeconomic, and biological factors. Climate change, urbanization, migration, and deforestation have significantly altered the habitats of sandflies, expanding the geographic range of disease transmission. Moreover, the emergence of treatment‐resistant *Leishmania* strains and co‐infections, such as HIV‐VL, have further complicated disease management and necessitated urgent innovation in diagnostic and therapeutic approaches. While liposomal amphotericin B and miltefosine have improved treatment outcomes, the high cost of these therapies and the potential for adverse side effects remain significant barriers to their widespread use in endemic regions [4–6].

In recent years, global initiatives such as the WHO Neglected Tropical Diseases Roadmap and regional elimination programs have prioritized the control of VL. However, achieving sustainable elimination requires addressing numerous gaps in our understanding of disease pathogenesis, host immune responses, and parasite biology. The development of vaccines, the identification of novel drug targets, and the implementation of integrated vector management strategies are critical to curbing the burden of VL. Furthermore, robust surveillance systems, community‐based health interventions, and investments in health infrastructure are essential to ensuring early diagnosis and treatment, particularly in remote areas where access to healthcare remains a challenge [7–9].

This review aims to provide a comprehensive synthesis of the current knowledge on VL, highlighting the major challenges in disease management, the latest innovations in diagnostics, therapeutics, and vector control, and the future directions necessary to achieve sustained control and eventual elimination. By focusing on a multidisciplinary approach, this work seeks to underscore the importance of collaborative efforts in addressing the multifaceted nature of VL, ultimately contributing to global health equity and the reduction of morbidity and mortality associated with this devastating disease [10–12].

## 2. Materials and Methods

This review employs a systematic approach to collate, synthesize, and analyze the existing literature on VL, focusing on its epidemiology, clinical manifestations, diagnostics, treatment modalities, and control strategies. The methodology aligns with the Preferred Reporting Items for Systematic Reviews and Meta‐Analyses (PRISMA) guidelines to ensure transparency, reproducibility, and comprehensiveness. A detailed description of the key steps involved in the review process is provided below. For vector control evidence, we specifically screened for insecticide susceptibility and molecular resistance surveillance studies in endemic countries to summarize the current status of sandfly resistance. For biological control, we identified studies reporting experimental virulence of entomopathogenic fungi (e.g., *B. bassiana* against *P. papatasi*) and molecular detection of Wolbachia in natural sandfly populations [10, 12].

The first step involved the formulation of a research question using the Population, Intervention, Comparison, Outcome, and Study Design (PICOS) framework. The primary research question was defined as follows: *What are the current challenges, innovations, and future directions in the management of visceral leishmaniasis globally?* This question guided the development of inclusion and exclusion criteria, which were applied throughout the review process. Studies were included if they provided data on any aspect of VL management, including epidemiology, clinical features, diagnostic advancements, therapeutic innovations, vector control, or public health interventions. Articles focusing exclusively on cutaneous or mucocutaneous leishmaniasis were excluded, unless their findings were relevant to the broader understanding of VL [11, 13].

The literature search was conducted across multiple electronic databases, including PubMed, Scopus, Web of Science, and Google Scholar, to ensure a comprehensive coverage of peer‐reviewed articles. The search strategy was designed to capture a wide range of studies by combining Medical Subject Headings (MeSH) terms and free‐text keywords related to VL. Examples of search terms included “visceral leishmaniasis,” “kala‐azar,” “Leishmania donovani,” “sandfly,” “vector control,” “treatment,” “diagnostics,” and “epidemiology.” Boolean operators (AND, OR) and truncation symbols were used to refine and broaden the search. The search was limited to articles published in English between January 2000 and December 2024 to focus on recent advances and current challenges [14, 15].

All search results were imported into reference management software (e.g., EndNote or Mendeley) for organization and duplicate removal. Two independent reviewers screened the titles and abstracts of retrieved articles for relevance. Full‐text articles were subsequently assessed against the inclusion criteria. Any discrepancies between the reviewers were resolved through discussion or consultation with a third reviewer to minimize bias. The quality of included studies was evaluated using standardized tools appropriate for each study design, such as the Newcastle–Ottawa Scale for observational studies and the Cochrane Risk of Bias Tool for randomized controlled trials [16–18].

Data extraction was conducted using a predefined data collection form, which captured information on study characteristics (e.g., authors, publication year, study location), research objectives, methods, key findings, and limitations. Extracted data were tabulated to facilitate thematic synthesis and identification of trends, gaps, and emerging patterns. Thematic analysis was employed to categorize findings into major themes, such as epidemiological trends, diagnostic tools, therapeutic options, and vector control strategies [19, 20].

To ensure methodological rigor, the review process also included an assessment of publication bias and heterogeneity. Funnel plots and Egger’s test were used to evaluate the presence of publication bias, while statistical tests such as *I* ^2^ were applied to assess heterogeneity among studies. Where applicable, a narrative synthesis was used to complement the quantitative analysis, providing context and depth to the findings [21, 22].

This review adhered to ethical standards by ensuring proper attribution to all original studies and avoiding data manipulation or selective reporting. The findings from this systematic review are expected to provide valuable insights into the challenges and opportunities in the management of VL, informing policy decisions, research priorities, and clinical practices [23, 24].

## 3. Results

The findings of this review offer a comprehensive analysis of the challenges, innovations, and future directions in the global management of VL. The results are presented under key thematic areas, highlighting critical advancements and persistent gaps in the understanding and control of this complex disease.

VL exhibits a diverse global distribution, with the majority of cases concentrated in South Asia, East Africa, and South America. In South Asia, India and Nepal continue to contribute the majority of the global VL burden, although Bangladesh achieved a historic milestone in 2023 by becoming the first country worldwide to eliminate VL as a public health problem [25]. Collectively, South Asia still accounts for more than two‐thirds of reported global cases, particularly in endemic pockets of India and Nepal. The disease is predominantly rural in nature, although urban transmission has been increasingly reported, particularly in regions affected by unplanned urbanization and migration. In East Africa, countries such as Sudan, Ethiopia, and Kenya face recurrent outbreaks driven by environmental changes, displacement of populations due to conflict, and inadequate healthcare infrastructure. In South America, Brazil accounts for the majority of VL cases, with the disease spreading to peri‐urban and urban areas due to deforestation and migration. Despite the implementation of national and regional control programs, the incidence of VL remains alarmingly high in many endemic areas. Surveillance data indicate significant underreporting of cases, particularly in remote regions where healthcare access is limited. Furthermore, climatic factors, including rising temperatures and changing rainfall patterns, have expanded the geographical range of sandflies, increasing the risk of transmission in previously non‐endemic areas [1, 2, 8].

The clinical presentation of VL varies across regions, with common symptoms including prolonged fever, hepatosplenomegaly, severe weight loss, and pancytopenia. Delayed diagnosis remains a critical issue, as the disease often mimics other febrile illnesses, leading to misdiagnosis and inappropriate treatment. Co‐infections with HIV pose a significant challenge, particularly in East Africa, where up to 30% of VL cases are associated with HIV. HIV‐VL co‐infection not only complicates diagnosis but also increases the risk of treatment failure, relapse, and mortality [26–28].

Recent innovations in VL diagnostics have focused on improving accuracy, accessibility, and cost‐effectiveness. The rK39 rapid diagnostic test (RDT) has become a cornerstone of VL diagnosis in many endemic regions due to its simplicity and high sensitivity. However, variability in test performance across regions and the potential for cross‐reactivity with other diseases, such as malaria, remain limitations. Molecular diagnostic tools, including polymerase chain reaction (PCR), offer superior sensitivity and specificity but are often limited to research settings due to their high cost and technical complexity. The development of point‐of‐care molecular diagnostics and the integration of multiplex assays represent promising advancements in this field [29–31].

The treatment landscape for VL has evolved significantly, with liposomal amphotericin B emerging as the gold standard in many regions. Single‐dose regimens have shown high efficacy and safety, particularly in South Asia. However, access to this treatment remains limited in resource‐poor settings due to its high cost and cold chain requirements. Miltefosine, the first oral drug for VL, has expanded treatment options, but concerns over teratogenicity and the emergence of drug resistance have highlighted the need for alternative therapies. Combination therapies, which leverage the synergistic effects of existing drugs, have shown promise in reducing treatment duration and minimizing resistance development [32–34].

Vector control remains a cornerstone of VL prevention, with indoor residual spraying (IRS) and insecticide‐treated nets (ITNs) being the most widely implemented strategies. However, the effectiveness of IRS and ITNs has been compromised in some settings by insecticide resistance and reduced susceptibility in sandfly populations. On the Indian subcontinent *Phlebotomus argentipes*, the principal vector of VL shows long‐standing resistance to DDT and evidence of reduced susceptibility or emerging resistance to pyrethroids (including deltamethrin, lambda‐cyhalothrin, and alpha‐cypermethrin) in several endemic districts, particularly in Bihar and neighboring areas; molecular surveillance has identified kdr mutations in the voltage‐gated sodium channel that are associated with DDT resistance and survival to low‐level pyrethroid exposure. In Sri Lanka, *P. argentipes* populations have shown reduced susceptibility to malathion in some sites, whereas earlier susceptibility surveys in Nepal and Bangladesh reported high mortality to pyrethroids and DDT in older studies though surveillance is intermittent and recent data indicate the need for continued monitoring. These findings underscore the importance of regular, standardized insecticide susceptibility monitoring, consideration of insecticide rotation or non‐chemical tools in integrated vector management, and molecular markers to inform resistance management strategies ([35–38]), as well as operational constraints in endemic regions. Recent research has explored the potential of biological control methods, such as the use of entomopathogenic fungi and Wolbachia‐infected sandflies, to complement traditional vector control, strategies. Recent laboratory work has demonstrated the potential of biological control approaches. For example, two local isolates of the entomopathogenic fungus *Beauveria bassiana* significantly reduced longevity of adult *Phlebotomus papatasi* in Iran under semi‐field conditions, indicating virulence against the vector. Additionally, molecular surveys have confirmed natural *Wolbachia* infections in field populations of several sandfly vectors including *Lutzomyia longipalpis* in Brazil, and *P. papatasi* and *P. perniciosus* in Spain and Morocco supporting the feasibility of exploring *Wolbachia*‐based strategies in leishmaniasis control [39–43]. Additionally, community‐based interventions, such as environmental management and education campaigns, have shown success in reducing sandfly breeding sites and human‐sandfly contact [44–46]. The effectiveness of IRS and ITNs has been compromised by insecticide resistance in sandfly populations. Studies have reported widespread DDT resistance in *Phlebotomus argentipes* in India and Nepal, with emerging evidence of pyrethroid tolerance. This necessitates the evaluation of alternative insecticides and integrated resistance management strategies [47–50].

Global and regional initiatives, including the WHO Neglected Tropical Diseases Roadmap and the Kala‐Azar Elimination Program, have played a pivotal role in mobilizing resources and fostering collaboration among stakeholders. However, the sustainability of these efforts is threatened by inadequate funding, political instability, and competing health priorities in endemic countries. Strengthening health systems, enhancing community engagement, and fostering partnerships between public and private sectors are critical to overcoming these challenges [51, 52].

The development of vaccines against VL remains a top priority in global health research. Several vaccine candidates have shown promising results in preclinical studies, but challenges related to efficacy, safety, and production scalability must be addressed before widespread deployment. Advances in genomic and proteomic technologies have provided new insights into the biology of *Leishmania* parasites, paving the way for the identification of novel drug targets and biomarkers. Furthermore, the integration of digital tools, such as mobile health applications and geographic information systems (GIS), offers opportunities to enhance disease surveillance, optimize resource allocation, and improve patient outcomes [53–55].

**Figure 1:**
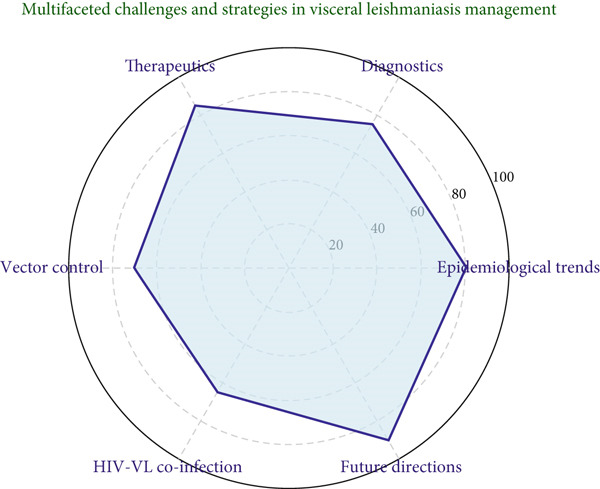
Comprehensive analysis of challenges and strategies in the management of visceral leishmaniasis.

**Figure 2:**
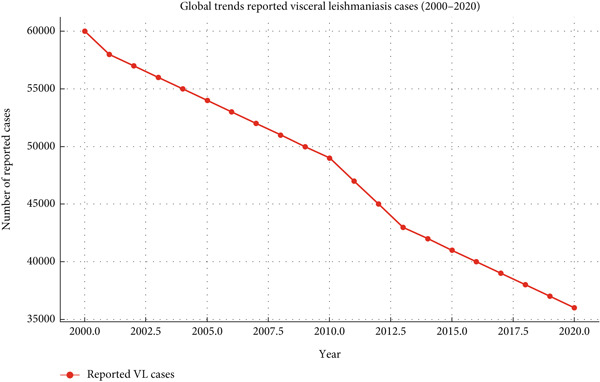
Global trends in reported visceral leishmaniasis cases (2000–2020). This figure illustrates the global trend of reported visceral leishmaniasis (VL) cases between 2000 and 2020. The data show a gradual but consistent decline over two decades, reflecting the impact of improved diagnostic tools, therapeutic interventions, and vector control strategies. While South Asia, East Africa, and South America have remained the most affected regions, intensified elimination programs particularly in South Asia have contributed significantly to reducing the global burden. Despite this progress, underreporting in remote areas and persistent outbreaks in East Africa highlight ongoing challenges in achieving sustainable elimination.

**Figure 3:**
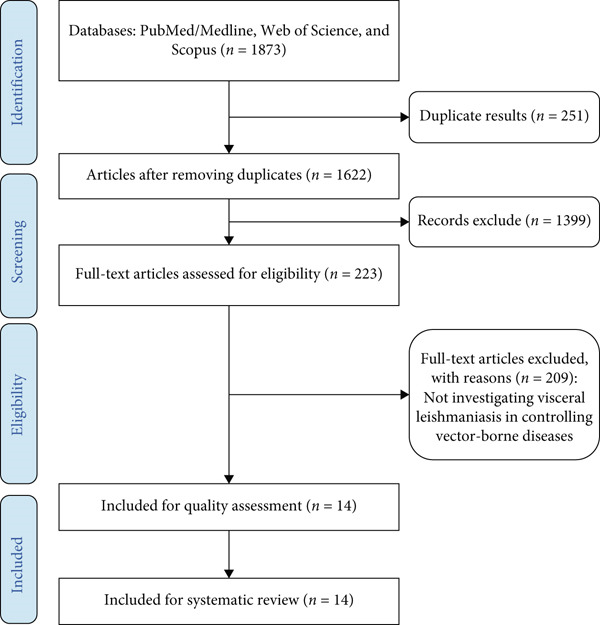
The PRISMA flow diagram.

This review highlights the multifaceted nature of VL and underscores the need for a multidisciplinary approach to address the complexities of its management. By consolidating the current state of knowledge and identifying key areas for intervention, this study aims to contribute to global efforts to reduce the burden of VL and move closer to its elimination (Tables 1 and 2 and Figures 1, 2, and 3).

**Table 1:** Overview of current therapeutic options for visceral leishmaniasis.

**Drug**	**Administration route**	**Dosage**	**Efficacy**	**Major side effects**	**Limitations**
Liposomal amphotericin B	Intravenous	3–5 mg/kg daily or single‐dose 10 mg/kg	> 95% (in India)	Fever, chills, nephrotoxicity	High cost; cold chain required
Amphotericin B deoxycholate	Intravenous	0.75–1.0 mg/kg/day for 15–20 days	90–95%	Nephrotoxicity, infusion reactions	Long duration; hospital admission needed
Miltefosine	Oral	2.5 mg/kg/day for 28 days	94% (India); lower in Africa	Vomiting, diarrhea, teratogenicity	Teratogenic; risk of resistance; contraindicated in pregnancy
Paromomycin	Intramuscular	15 mg/kg/day for 21 days	~ 95% (India)	Ototoxicity, nephrotoxicity	Limited availability; injectable
Sodium stibogluconate (SSG)	Intramuscular/IV	20 mg/kg/day for 30 days	Declining (Africa: < 80%)	Cardiotoxicity, pancreatitis	Resistance widespread in India and Sudan
Combination therapy (e.g., AmB + miltefosine)	Multiple	Varies by regimen	> 95%	Depends on drugs used	Requires programmatic implementation

**Table 2:** Comparative analysis of diagnostic tools for visceral leishmaniasis.

**Diagnostic tool**	**Type**	**Sensitivity**	**Specificity**	**Advantages**	**Limitations**
rK39 Rapid Diagnostic test	Serological (immunochromatographic)	95–100% (India), 70–85% (East Africa)	90–95%	Easy to use; field‐friendly; fast results	Reduced accuracy in HIV co‐infection; regional variability
Direct agglutination test (DAT)	Serological	90–99%	90–95%	High sensitivity; applicable to stored sera	Requires cold chain; complex protocol
Microscopy (splenic/bone marrow aspirate)	Parasitological	93–99% (splenic); 53–86% (bone marrow)	100%	Direct visualization of parasite	Invasive; requires skilled personnel
PCR (e.g., kDNA, ITS1)	Molecular	> 95%	> 95%	High sensitivity and specificity; species detection	Expensive; requires lab infrastructure
Loop‐mediated isothermal amplification (LAMP)	Molecular	85–95%	90–97%	Rapid; field‐adaptable in some formats	Still under evaluation for routine use
Leishmania antigen ELISA	Antigen detection	85–95%	90–95%	Non‐invasive (urine‐based); emerging tool	Limited availability; requires further validation

## 4. Discussion

VL remains a significant global health challenge, affecting some of the most vulnerable populations in endemic regions. Despite notable advancements in diagnostics, treatment, and vector control, persistent gaps in knowledge and implementation hinder efforts to achieve sustainable control and eventual elimination. This discussion synthesizes the findings presented in this review, critically evaluates current strategies, and explores potential pathways for improving the management of VL on a global scale [1, 8, 56]. The success of Bangladesh highlights the feasibility of regional elimination programs through sustained political commitment, vector control, and community‐based interventions, offering a model for other endemic countries in South Asia and East Africa [25, 57, 58]. These findings support further investigation of *B. bassiana* formulations and the feasibility of Wolbachia‐based vector modification approaches, as part of integrated vector management for VL.

The complex epidemiology of VL reflects the interplay between environmental, socioeconomic, and biological factors. Climate change, urbanization, and human migration are reshaping the geographic distribution of VL, introducing new challenges for surveillance and control programs. In regions such as South Asia and East Africa, where VL is endemic, these shifts exacerbate the vulnerability of already underserved populations. Strengthening surveillance systems is crucial to detecting and responding to outbreaks in a timely manner. Integrating geographic information systems (GIS) and predictive modeling into public health frameworks could enhance the precision of disease mapping and facilitate resource allocation. A critical challenge lies in underreporting, particularly in remote and conflict‐affected areas where healthcare access is limited. Efforts to improve health infrastructure and integrate VL surveillance with other disease monitoring systems could address this issue. Additionally, fostering community engagement through education and participatory approaches can empower local populations to contribute to early detection and prevention efforts [59, 60].

Advancements in diagnostics have significantly improved the ability to detect VL, yet regional disparities in test performance highlight the need for context‐specific solutions. The rK39 rapid diagnostic test, widely used due to its affordability and simplicity, has demonstrated high sensitivity in South Asia but variable results in East Africa and other regions. This underscores the importance of developing regionally tailored diagnostic tools that account for genetic and antigenic diversity in *Leishmania* strains. Molecular diagnostics, such as PCR, represent a gold standard in accuracy but remain inaccessible in most endemic areas due to cost and logistical challenges. Innovations in point‐of‐care molecular technologies, including loop‐mediated isothermal amplification (LAMP), offer promising alternatives by combining high sensitivity with operational simplicity. Expanding access to these tools through partnerships with industry and international health organizations is essential to overcoming diagnostic bottlenecks [29, 31, 61].

Treatment advancements, particularly the introduction of liposomal amphotericin B and miltefosine, have improved patient outcomes. However, the high cost and logistical demands of these therapies limit their availability in resource‐constrained settings. Single‐dose regimens of liposomal amphotericin B have shown promise in South Asia but are less effective in East Africa, where higher parasite loads and differing host immune responses necessitate alternative approaches. Combination therapies, which leverage synergistic effects to enhance efficacy and reduce treatment duration, represent a critical area for further research. Drug resistance is an emerging threat that could undermine current treatment strategies. Reports of resistance to miltefosine, coupled with concerns over its teratogenicity, highlight the urgent need for new therapeutic agents. Advances in genomic and proteomic research have identified novel drug targets, but translating these discoveries into clinically viable treatments requires sustained investment in drug development pipelines. Additionally, integrating pharmacovigilance into national health programs could help monitor resistance patterns and inform treatment guidelines [33, 62, 63].

Vector control remains a cornerstone of VL prevention, yet its effectiveness is increasingly compromised by operational challenges and insecticide resistance. Indoor residual spraying (IRS) and ITNs have achieved notable success in reducing sandfly populations, but their long‐term sustainability depends on addressing resistance and ensuring consistent implementation. Research into alternative insecticides, rotation strategies, and synergists that enhance efficacy is vital to maintaining the effectiveness of chemical interventions [64–67]. Innovative approaches, such as the use of Wolbachia‐infected sandflies and entomopathogenic fungi, offer potential for sustainable vector management. However, these methods require rigorous field trials to evaluate their effectiveness, scalability, and ecological impact. Community‐based interventions, including environmental management and education campaigns, also play a critical role in reducing vector breeding sites and human‐vector contact. Empowering communities to take ownership of vector control activities can enhance their effectiveness and ensure long‐term sustainability [44, 45, 68].

HIV‐VL co‐infection presents a formidable challenge, particularly in regions where both diseases are endemic. Co‐infection exacerbates immunosuppression, complicates diagnosis, and increases the risk of treatment failure and relapse. Integrated approaches that address both diseases simultaneously are essential to improving outcomes for co‐infected patients. Expanding access to antiretroviral therapy (ART) and ensuring early detection and treatment of VL in HIV‐positive individuals could significantly reduce morbidity and mortality [26, 69, 70].

Achieving sustainable control and elimination of VL requires a multidisciplinary approach that addresses gaps in knowledge, resources, and infrastructure. Key priorities include, while several vaccine candidates are in development, challenges related to efficacy, safety, and production scalability must be addressed. Collaborative efforts between academia, industry, and global health organizations are essential to accelerating vaccine research and deployment (Vaccine Development), Combining diagnostics, therapeutics, and vector control strategies into cohesive programs could enhance the effectiveness of interventions and reduce duplication of efforts (Integrated Approaches), Leveraging digital tools, such as mobile health applications and GIS, could improve disease surveillance, optimize resource allocation, and enhance patient care (Innovative Technologies), Investing in local research capacity and healthcare infrastructure is crucial to empowering endemic countries to lead VL control efforts (Capacity Building) [53, 54, 71].

## 5. Conclusion

The management of VL has advanced significantly, yet critical challenges remain. Addressing these challenges requires sustained commitment, innovative solutions, and collaborative efforts across disciplines and sectors. By building on current knowledge and embracing emerging technologies, the global community can move closer to the goal of eliminating VL and alleviating the suffering it causes [52, 72].

## Ethics Statement

The author has nothing to report.

## Consent

The author has nothing to report.

## Conflicts of Interest

The author declares no conflicts of interest.

## Author Contributions

E.A. has carried out all parts of the article, including design, execution, and writing.

## Funding

No funding was received for this manuscript.

## Supporting Information

Additional supporting information can be found online in the Supporting Information section.

## Supporting information


**Supporting Information 1**  

**Supporting Information 2**  

## Data Availability

All data supporting the findings of this study are available within the article. Additional information or extracted datasets used during the systematic review are available from the corresponding author upon reasonable request.
