# Supplementary material for: Advancing Insights Into Visceral Leishmaniasis: Challenges, Innovations, and Future Directions in Global Disease Management
Source: J Parasitol Res. 2025 Dec 30;2025:5233179. doi: 10.1155/japr/5233179 (PMC12767020; doi:10.1155/japr/5233179)
Supplement: Supplementary file 2 — Supporting Information 2 [file JAPR-2025-5233179-s002.docx]

***Highlights***

- Provides a comprehensive review of global challenges in the management of visceral leishmaniasis (VL).
- Examines recent advancements in diagnostics, including rK39 tests and molecular approaches.
- Analyzes therapeutic innovations like liposomal amphotericin B and combination therapies.
- Explores the impact of climate change and urbanization on VL epidemiology and spread.
- Discusses vector control strategies, including biological methods and community-led initiatives.
- Highlights the burden of HIV-VL co-infection and integrated approaches for improved outcomes.
- Proposes future directions such as vaccine development, digital tools, and inter-sectoral collaborations.
